# The informativeness of the Johns-matrix image of laser autofluorescence in blood plasma films for early diagnosis of endometriosis associated with infertility

**DOI:** 10.25122/jml-2022-0185

**Published:** 2022-12

**Authors:** Oksana Valerianivna Bakun, Nataliia Sergiivna Voloshynovych, Yevheniia Anatoliivna Dudka, Nataliia Vasylivna Lisnianska, Oksana Volodymyrivna Kolesnik, Ksenia Valeriivna Slobodian, Alina Pradatu

**Affiliations:** 1 Obstetrics and Gynecology Department, Bukovinian State Medical University, Chernivtsi, Ukraine; 2 Department of Physiology J. D. Kirschenblatt, Bukovinian State Medical University, Chernivtsi, Ukraine; 3 Department of Pathological Physiology, Bukovinian State Medical University, Chernivtsi, Ukraine; 4 Materno-Fetal Assistance Excellence Unit, Polizu Clinical Hospital, Alessandrescu-Rusescu National Institute for Mother and Child Health, Bucharest, Romania

**Keywords:** autofluorescence, biopsy, plasma, infertility

## Abstract

We used Jones-matrix images of protein networks of blood plasma films obtained from laser autofluorescence and determined the sensitivity, specificity, accuracy, and prognostication of positive and negative results. Our study aimed (1) to develop and substantiate new approaches for the diagnosis of endometriosis by improving the methods of Jones-matrix mapping of laser-induced auto-fluorescence and (2) to develop statistical approaches for analyzing the distribution of values of the true component of Jones-matrix images of blood plasma. Biological preparations were performed for two groups: 35 samples from control group 1 (women with infertility of unknown origin) and 85 samples from group 2 with endometriosis (women with infertility and endometriosis). The strength of the Jones-matrix method of autofluorescence mapping of plasma proteins taken from both groups was maximal for the decisions determined based on the calculation of the statistical moment of the 4^th^ order, for statistical moment Z4, characterizing the sharpness of the peak distribution of the polycrystalline component of the plasma film. Comparison with the similar informative data of the Jones-matrix laser autofluorescence method of histological sections of the endometrial biopsy under conditions of blind endometriosis diagnosis revealed this method of analysis highly informative. Therefore, this technique can be used in screening studies to form a risk group.

## INTRODUCTION

The scattering of optical radiation by biological objects and media is considered in approximating the statistical averaging of photometric and polarization parameters. The most widespread diagnostic methods are based on studying scattered radiation fields employing classical photometry, Stokes polarimetry, and Muller matrix optics [1]. In parallel to these scientific directions, laser methods for studying optically inhomogeneous biological structures – correlation optics and speckle optics, which use coherent polarized radiation as a probe, were developed. The following main results can be distinguished from these studies. First, this is the relationship between a set of statistical moments that characterize the opto-geometric structure (distribution of optical axis directions and birefringence) of biological crystals of the extracellular matrix of the three basic types of biological tissues of humans and statistical (mean, dispersion, excesses) and correlation (half-width, variance of fluctuations, radius of correlation of auto- and inter-correlation functions) parameters of coordinate distributions of elements and phase angles of Jones matrix [2]. Based on this, the transformation mechanisms of the amplitude-phase parameters of laser radiation by a set of optically uniaxial birefringent crystals of the extracellular matrix of the three main types of histological sections [3] of biological tissues (epithelial, muscular, and connective) which fully characterize the polarization-phase properties in human are investigated and analyzed. Subsequently, scenarios of the formation of coordinate distributions of elements of the Jones matrix by different types of histological sections of biological tissues were determined, and a system of classification of their optical properties was developed by estimating ranges of change of values of statistical moments of the 1^st^–4^th^ orders of distributions of real parts of a set of matrix elements [4].

The physical reason for the formation of two-dimensional distributions of the Jones matrix elements is the coordinate modulation of phase shifts between the orthogonal components of the laser wave amplitude [5], which interacts with the statistically distributed two-ray biopsy of biological crystals of the extracellular matrix of the main types of histological sections of biological tissues. The result revealed a relationship between average, variance, asymmetry, and excesses, which characterize the orientation (distribution of optical axis directions) and phase (distribution of phase shifts) structure of the optically anisotropic grid of histological sections of biological tissues and the totality of the coordinates of the organisms. The increase in the dispersion of the optical axes directions of biological crystals is manifested by the increase of asymmetry and the decrease of excess of two-dimensional distributions of real parts of the Jones matrix elements of epithelial, muscular and connective tissue [6].

The growth of phase shift dispersion due to extracellular matrix anisotropy is accompanied by reversed changes in the statistical moments of the 3^rd^ and 4^th^ orders. On this basis, the principles of Jones-matrix classification of the polarization-phase properties of the basic types of histological sections of biological tissues according to the ranges of change of a set of statistical moments characterizing the corresponding matrix elements are established and substantiated.

In addition, the relationship between optical (anisotropy of biological crystals) and geometric (hierarchical structure of birefringent fibrils) has been experimentally discovered and theoretically substantiated.

The increase in the dispersion of orientations and the double-refraction of biological crystals is the physical reason for the decrease in the half-width of the correlation function and the increase in the dispersion of its fluctuations [7]. Therefore, the differentiation criteria for the polarization-phase properties of epithelial and connective tissues have been identified and substantiated.

Based on the correlation structure of two-dimensional distributions of real parts of Jones matrix elements, a physically substantiated relationship between the ranges of change in the radius of polarization correlation and the variance of fluctuations of interrelated functions of the 1^st^–4^th^ orders with a set of statistical moments (dispersion, asymmetry, asymmetry) characterize the distributions of the directions of the optical axes and the birefringence of biological crystals of the extracellular matrix of the three main types of biological tissues of man. As a result, the optical properties of the anisotropic extracellular matrix of all major types of histological sections of human biological tissues are differentiated for the first time.

The analytical relationship between local orientations of optical axes, the birefringence of biological crystals of extracellular matrix, and values of phase angles of elements of the Jones matrix were determined, which allowed establishing the physical reasons for changing the values of phase statistical moments of the distribution of imaginary parts of the histological elements of the matrix [8]. The growth of asymmetry and excess distributions of imaginary parts of matrix elements is caused by the increased dispersion of the directions of optical axes of biological crystals [9]. The change in optical anisotropy is manifested in reverse processes. On this basis, a phase tomography method of the opto-geometric structure of the extracellular matrix from histological sections of biological tissues of different morphological structures and physiological state was developed.

Differentiation of polarization-phase properties of complex (superposition of biological crystalline nets of different types of histological sections of biological tissues) extracellular matrix of organ tissues (liver, spleen, wall of the small and large intestine) of a person by autocorrelation analysis of coordinate distributions was realized. The proposed method was based on established and physically substantiated mechanisms [10] of correlation parameters of autocorrelation functions of phase angles as a result of phase modulation of laser radiation by a set of hierarchical structures for constructing birefringent fibrils. On this basis, preclinical diagnostics of the septic condition of organ tissues was first implemented.

On the other hand, there is a wide range of less studied biological objects. First, they include a variety of fluids – blood, bile, synovial fluid, liquor, and others. Therefore, further development of new approaches for the analysis of the vector structure of laser radiation fields, transformed not only by optically anisotropic layers of biological tissues but also by films of biological fluids, particularly their polycrystalline protein networks, is relevant [11].

The processes of transforming dendritic polycrystalline networks into spherulite with azimuthal symmetry of the directions of the optical axes of biological crystals are in opposite changes of the statistical, correlation, and spectral moments of the 3^rd^ and 4^th^ orders, which characterize the real component of Jones-matrix images [11]. The optical axis diagnostics of the formation of a cluster network of crystals ordered by the directions was realized, which is manifested in the equally probable coordinate distributions of the real component of the "phase" elements of the Jones matrix due to the increase in the depth of phase modulation of laser radiation. On this basis, a diagnosis of gallstone disease was implemented.

A scale-selective analysis of statistical, correlation, and spectral parameters was applied for the first time to classify and differentiate the optical properties of polycrystalline networks with weak phase modulation, which objectively characterize the distribution of the characteristic values of the imaginary component of the "Jones" element of the biologic film matrix [12]. As a result, the physical criteria for the differentiation of polarization manifestations of optical anisotropy of dendritic networks with a slight birefringence of partial biological crystals were obtained.

The relationship between the statistical, correlation, and spectral moments of the 1^st^–4^th^ orders, which characterize the sets of wavelet coefficients of dependence of the number of characteristic values of Jones-matrix images, and the distributions of the optical axes directions and birefringence of biological crystals on different scales determined dendritic network of saliva layers with weak phase fluctuations. On this basis, the diagnosis of tuberculosis was made.

In the following years, the diagnostic effectiveness of this method has been studied by many scientific centers [13]. At the same time, only two foreign publications describe the possibilities of autofluorescence detection of metaplasized epithelium of the mucous esophageal-gastric junction.

At certain stages of development, the autofluorescence spectra of malignant tumors show a red fluorescence of endogenous porphyrins produced by hemolytic processes or some types of bacteria capable of producing proto- and coproporphyrins [14].

Fluorescence bands recorded in the red region of the autofluorescence spectrum in this patient fully correspond to the fluorescence bands of endogenous protoporphyrin IX upon excitation of its fluorescence at 405 nm and 532 nm.

Thus, this observation showed that in the case of submucosal growth of cervical cancer, it is low differentiated [15].

In the esophageal adenocarcinoma, endogenous protoporphyrin IX accumulates, and its fluorescence is detected not only in the area of the tumor lesion [16] but also above the tumor in the visually unchanged mucosa of the esophagus.

The autofluorescence spectrum of biological tissues is a superposition of the fluorescence spectra of individual endogenous fluorophores and depends on many factors. Its intensity and shape are influenced by the concentration and distribution of endogenous fluorophores [17], their spectral characteristics of excitation and fluorescence, especially the metabolism, architectonics, and optical properties of the tissue, due to the presence of non-fluorescent chromophores.

The most significant endogenous fluorophores of the visible spectrum are:

- Connective tissue elements – collagen and elastin;
- Components of the respiratory chain – flavins and nicotinamide nucleotide (NADN), involved in redox processes;
- Porphyrins are involved in cell biosynthesis or are products of the biosynthesis of saprophytic or pathogenic microflora in bacterial infection [18].

With the development of neoplasia, epithelial tissues undergo various biochemical and structural changes reflected in the autofluorescence spectrum.

The differences in the autofluorescence spectra between the normal and dysplastic esophagus epithelium have been shown in several foreign publications. As our studies have shown, normally flat and metaplasmic cylindrical epithelium also have differences in the AF spectra [19].

Differences in the AF spectra of the normal planar and cylindrical epithelium with a metaplasmic intestinal cylindrical epithelium of the esophageal-gastric junction were detected upon excitation in the green region of the spectrum in 13 of 15 patients. An informative parameter is the ratio of integral intensities in the red and yellow regions of the spectrum [20], which significantly increases with the development of intestinal metaplasia. A sharp increase in this parameter is observed in esophageal adenocarcinoma. These data are in agreement with the results obtained in the study, which showed that the informative parameter characterizing differences in the AF spectra of the normal planar and metaplasied cylindrical epithelium is the intensities in the red and green regions of the spectrum when excited in the UV region [21]. Accordingly, the authors suggested that the transformation of metaplasied epithelium toward dysplasia III and esophageal adenocarcinoma is accompanied by an increase in this ratio [22].

Thus, the use of fluorescence spectroscopy (LFS) esophagogastroduodenoscopy and the evaluation of laser-induced autofluorescence of the gastrointestinal junction epithelium allows *in vivo* to obtain diagnostic information in real-time.

## MATERIAL AND METHODS

This section describes the study groups, schemes of experimental locations, and methods of measuring the parameters of polarizing laser autofluorescence by layers of human blood plasma. The basic analytical algorithms for statistical analysis of Jones-matrix images of polarizing laser autofluorescence are presented. Biological preparations of blood plasma films from two groups were used as objects in this experimental study:

- Control group 1 (women with infertility of unknown origin – 35 samples);
- Group 2 with endometriosis (women with infertility and endometriosis – 85 samples).

A drop of liquid was applied to an optically homogeneous glass and dried for 24 hours at room temperature.

### Basic model ideas about the optical properties of layers of biological tissues

As shown in numerous studies focused on biomedical optics, the following operator of the Jones matrix is the most adequate to describe the polarization properties of a layer of biological tissue [23].

{D}=‖d_11_ d_12_ d_21_ d_22_‖= =||p+p exp exp(-iδ) cos cos p sin sin p[1-exp exp(-iδ)] cos cos p sin sin p[1-exp exp(-iδ)]p+p exp exp (-iδ)||

The value of each element of the linear matrix operator is completely determined by two main parameters:

- ρ is the orientation direction of the optical axis of the crystal, which is determined by the direction of folding of the protein fibrils;
- δ is the magnitude of the phase shift introduced by two orthogonal amplitude components due to the double-refraction of the protein fibril.

It should be noted that such simulation is based on the assumption that an optically uniaxial crystal is approximated within the optical dimensions of the extracellular matrix elements.

On the other hand, devoted to the diagnostics of the processes of formation of the structure of biological tissues, a more complex (multidimensional) scenario of the formation of an extracellular matrix consisting of simpler fibers, fibers of the collagen type of protein and elastin, were analyzed [24].

Six different structural levels are distinguished in connective tissue. The first level (the simplest) is a collagen molecule; the sixth level is the connective tissue. Between them, in ascending order, are microfibrils, subfebriles etc.

Microfibrils are combined into the above-organized structures – subfebriles, which are rotating super-spirals. Furthermore, the fibril, in turn, consists of a set of probably located subfebriles.

The analog for supramolecular formations is the liquid crystal analog for biological tissues.

The term "liquid crystal" should be defined as an organized liquid characterized by the orderly nature of the units retained by the forces of attraction. In a certain temperature range and concentrations, "liquid crystals" are similar to elongated molecules oriented in one, two, or three dimensions [25].

Nematic liquid crystals are characterized by elongated molecules that lie parallel to each other in the absence of a multilayer organization, and therefore the ordering is only orientational.

In cholesteric liquid crystals, the molecules are arranged parallel to each other in each plane, and each plane is placed at a certain (constant) angle to the next plane of the crystals [26]. Therefore, this model of "liquid crystals" can be used to describe biological systems that obey the laws of transparent crystalline structures but have lost their liquid character.

### Jones optical diagram – matrix mapping of polarizing laser autofluorescence of biological tissues

**Figure 1:**
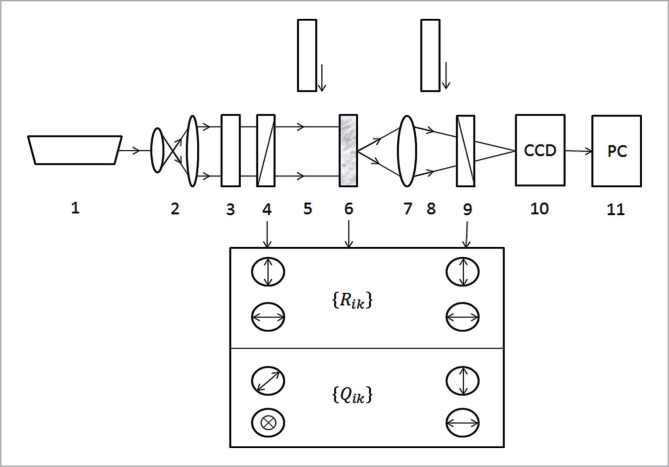
Optical diagram of a microscopic polarimeter, where 1 is a He-CD laser; 2 – collimator; 3 – stationary quarter-wave plate; 5, 8 – mechanically moving quarter-wave plates; 4, 9 – polarizer and analyzer respectively; 6 – object of study; 7 – micro lens; 10 – CCD camera with clipping interference filter; 11 is a personal computer.

Figure 1 shows an optical diagram of a microscopic polarimeter for measuring the set of coordinate distributions of the Jones matrix of laser autofluorescence of histological sections of the endometrium and films of biological fluids.

Excitation of laser autofluorescence was performed by illumination carried out by a parallel (∅=104 mkm) beam of a blue He-Cd laser (wavelength λ=0.414 mkm, power W=5.0 mW). The polarizing illuminator consists of quarter-wave plates 3; 5 and the polarizer 4, forming a laser beam with an arbitrary polarization state [27].

The polarization images of laser autofluorescence layers of biological tissues and liquids using a polarizing micro lens 7 (4× magnification) were projected into the photosensitive plane (800×600 pixels) of the CCD camera 10, which provided a range of measurement of structural elements of the image of biological 2 m fluids.

The experimental conditions were chosen to allow the spectral distribution of laser autofluorescence intensity to be spectrally separated by a clipping interference light (spectral transmission at a wavelength of 550 nm±20 nm).

Analysis of the polarization structure of images of laser autofluorescence histological sections of the endometrium and smears of biological fluids was carried out using a polarizer 9 and a quarter-wave plate 8.

### Methods of experimental determination of elements of the Jones matrix of laser autofluorescence of biological layers

In general, the Jones matrix operator consists of a set of four complex elements {A}=‖a_11_ a_12_ a_21_ a_22_‖;, which fully characterizes the poetic properties of the biological layer.

Typically, the operator {A} record in the form of two components – real {R}=‖R_11_ R_12_ R_21_ R_22_‖; and imaginary {Q}=‖Q_11_ Q_12_ Q_21_ Q_22_‖;.

The real component describes the ability of the biological layer to absorb laser radiation and the formation of laser autofluorescence [28]. The imaginary component describes the processes of phase modulation of laser radiation and, in this sense, is not relevant.

In our work, we applied a standard technique for measuring the true constituent elements of the Jones matrix.

1. We irradiated a layer of biological tissue or fluid linearly polarized beam of light of unit intensity with azimuth;

1.1. Then we passed the beam through polarizer 9 (Figure 1), the transmission plane of which was horizontal Θ=0°, measured the intensity I_1_=R_11_^2^;

1.2. The beam passing through the biological layer 6, allowed us to pass through the polarizer 9, the transmission plane of which was vertical Θ=90° and measure the intensity I_2_=R_21_^2^.

2. The biological layer 6 (Figure 1) was irradiated by a beam of linearly polarized laser light of unit intensity with azimuth

2.1. We passed the transformed laser beam through polarizer 9 (Figure 1), the transmission plane of which was horizontal Θ=0°, and measured the intensity I_3_=R_12_^2^.

2.2. We turned the polarizer 9 to the angle Θ=90° and measured the intensity I_4_=R_22_^2^.

3. We calculated for each individual pixel of the digital camera 10 (Figure 1) a set of four real constituent elements of the Jones laser polarizing autofluorescence matrix by the following algorithm: {R_11_(r)=√I_1_; R_12_(r)=√I_2_; R_21_(r)=√I_3_; R_22_(r)=√I_4_. (2.2)

4. We found the coordinate distribution of the elements of the Jones matrix laser polarization autofluorescence within the entire set of pixels (m×n) photosensitive pad digital camera 10 (Figure 1).


$$R_{ik}=\left[{\left(R_{ik}\right)}_{11}\to \to \;{\left(R_{ik}\right)}_{1m}↓↓↓↓\;{\left(R_{ik}\right)}_{n1}\to \to \;{\left(R_{ik}\right)}_{nm}\right].\left(2.3\right)$$

### Basic algorithms for the statistical analysis of coordinate distributions of elements of the Jones matrix of laser autofluorescence of biological layers

**Figure 2:**
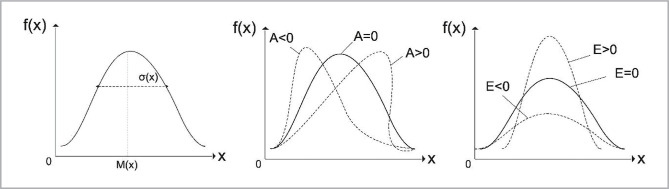
Statistical moments of the 1^st^–4^th^ orders (mean, variance, asymmetry, excess).

To estimate the coordinate distributions of random values of coordinate distributions R_ik_ (m×n) their histograms are determined. Then the set of their statistical moments of the first – fourth orders was calculated (Figure 2).

By statistical momentum of the first order, we mean the average value of the random values of the coordinate distributions of Jones-matrix elements.


$$M_{1}=\frac{1}{N}\sum_{i=1}^{N}\left|{\left(R_{ik}\right)}_{i}\right|\text{ (2.4)}$$

Under the variance (second-order statistical moment) of a random variable, we will understand the degree of variation of a given random variable, that is, its deviation from the mathematical expectation.


$$M_{2}=\sqrt{\frac{1}{N}\sum_{i=1}^{N}{\left(R_{ik}\right)}_{i}{}^{2}}\text{ (2.5)}$$

Asymmetry is called a value that characterizes the deviation from the normal distribution of a random variable.


$$M_{3}=\frac{1}{M_{2}{}^{3}}\frac{1}{N}\sum_{i=1}^{N}{\left(R_{ik}\right)}^{3}\text{ (2.6)}$$

The asymmetry coefficient is positive if the right tail of the distribution (Figure 2) is longer than the left one and is negative otherwise.

The excess will mean the severity of the "peak" of the distribution of a random variable.


$$M_{4}=\frac{1}{M_{2}{}^{4}}\frac{1}{N}\sum_{i=1}^{N}{\left(R_{ik}\right)}_{i}{}^{4}\text{ (2.7)}$$

N means the full number (800×600) of the pixels of the CCD-camera 10 (Figure 1).

### Principles and methods of evidence-based medicine in the Jones method – matrix mapping of laser autofluorescence of biological tissues and fluids

This section provides materials that highlight the main approaches to evidence-based medicine in the objective analysis of a set of statistics (statistical moments of the 1^st^–4^th^ order, characterizing the histograms of the elements of the Jones matrix of laser autofluorescence of biological tissues and liquids taken from groups 1 and group 2.

### General principles of medical diagnostics using Jones matrix microscopic images of biological samples

All optical-physical methods that operate with the concept of image for medical diagnostics can be divided into two groups:

1. Hardware-dependent diagnostic systems – their result depends on the physical-technical factors of obtaining the medical image and a set of objective parameters that characterize its structure;
2. Operating Dependent Diagnostic Systems – medical image analysis is related to the subjective diagnosis of a physician who examines a biological drug.

Ideally, one should have such a diagnostic system for the study of a biological drug in order to obtain the necessary and reliable diagnostic information based on a set of objective criteria that characterize the structure of Jones-matrix images of the laser autofluorescence of the biological layer to make the right clinical decision [29]. At the same time, it is necessary to make maximum use of scientific data presented in authoritative and leading journals with an impact factor and included in scientific metric databases (SCOPUS, Google scholar etc). Such publications are based on the generalizability of the research data using adequate model ideas and objective criteria, independent of the subjectivity of diagnosis [30]. In addition, one must use randomized groups of biologicals and check diagnostic methods with the standard method of diagnosis – the gold standard.

### Basic principles of evidence-based medicine in the objective analysis of Jones-matrix images of laser autofluorescence biological products

The main prerequisites for applying the principles of evidence-based medicine are:

- Probabilistic meaning of medical diagnosis, obtained from the study of Jones-matrix images of laser autofluorescence of biological products;
- Bias and subjectivity of diagnostic studies and expert findings regarding the state of the biological preparation;
- The presence of random errors in equipment and computer analysis of the structure of Jones-matrix images of laser autofluorescence of biological products.

The optimal or reliable situation occurs when the diagnostic technique is based on scientifically sound and experimentally confirmed interrelation of polycrystalline construction and reliable information on the structure of Jones matrix images of laser autofluorescence of biological products.

The objective (statistical) parameters of the distribution of Jones-matrix images of laser autofluorescence biologicals should be based on modern, generally recognized approaches and stimulate the skill of the physician-diagnostician.

### The strength of the method of Jones-matrix images of laser autofluorescence of biological products

In evidence-based medicine, "the power of the method" determines the success rate of a diagnostic test based on a comprehensive statistical analysis of Jones-matrix images of laser autofluorescence biologicals to influence medical decision-making.

Important criteria characterizing the methods of Jones-matrix images of laser autofluorescence biological products are the reliability, reproducibility, and convergence of the study results.

The validity of the method shows to what extent the resulting set of statistics characterizing the Jones-matrix images of laser autofluorescence biologicals corresponds to the patient's specific condition, determined by the gold standard [31].

When conducting our research, samples of biologicals taken from different patient groups would use the commonly accepted terminology of evidence in medicinal medicine:

The interpretation of "positive" for patients with the presence of the disease. This is a "true positive case".

1. The interpretation of "negative" for patients with no disease. This is a "true negative" – (TN);
2. The interpretation of "positive" for patients with no disease. This is a "false positive" – (FP);
3. The interpretation of "negative" for patients with the presence of the disease. This is a "false negative" – (FN).

### Characteristics of informativeness of Jones-matrix images of laser autofluorescence of biological preparations

To characterize the informative nature of any diagnostic method, objective parameters called operational characteristics are used [32]. There are basic and auxiliary characteristics. The main characteristics include:

- Sensitivity (Se) – is the proportion of correct positive results (TP) of the diagnostic method among all patients (D_+_)
$$Se=\frac{TP}{D_{+}}100\%\text{ (2.8)}$$
- The greater the sensitivity of the test, the more often the disease will be detected. This fact indicates the effectiveness of the method. In this sense, they are identifiers.
- Specificity (Sp) – is the proportion of correct negative results (TN) of the technique among a group of healthy patients (D___)
$$Sp=\frac{TN}{D_{-}}100\%\text{ (2.9)}$$
- Based on the SP definition, we can identify the proportion of healthy patients for whom the analysis of Jones-matrix images of laser autofluorescence of biological products gives a negative result. Highly specific diagnostic tests are called discriminators.
- The second group of informative indicators refers to the following supporting criteria:
- Accuracy (Ac) – proportion of correct results (TP+TN) test among all patients examined (D_+_+D___)
$$Ac=\frac{TP+TN}{D_{+}+D_{-}}100\%\text{ (2.10)}$$
- The accuracy, therefore, reflects the number of correct diagnoses obtained by testing the Jones-matrix method of laser autofluorescence of biological agents.

For the proper understanding of the diagnostic effectiveness of this method of study, probabilistic criteria that determine the probability of the disease (or lack thereof) in the known result of the study play an important role [33].

Positive Predictability (+VP) is the ratio of correctly (true) positive cases (TP) to all positives (TP+FN) from the method of Jones-matrix images of laser autofluorescence of biological products


$$+VP=\frac{TP}{TP+FN}100\%\text{ (2.11)}$$

- The prognosis of a positive result directly indicates how high the likelihood of illness is with positive study results.
- The predictability of a negative result (-VP) – is the ratio of true negative cases (TN) to all negative decisions (TN+FP). This criterion is determined by the formula

$$-VP=\frac{TN}{TN+FP}100\%\text{ (2.12)}$$

This criterion indicates how likely the patient is to be healthy if the results of the Jones-matrix method of laser autofluorescence of biological drugs are negative.

## RESULTS

**Figure 3:**
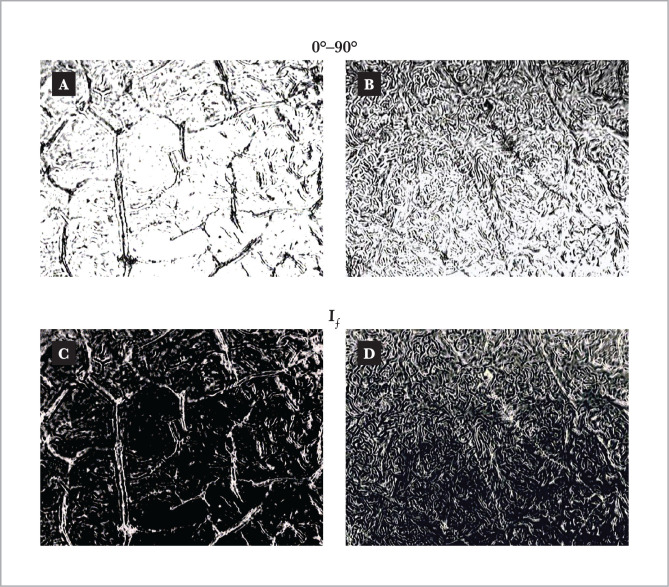
Polarization - visualized () and autofluorescence laser microscopic images of polycrystalline networks of blood plasma film proteins of two groups: (group 1) – norm [fragments (a), (c)]; (group 2) - endometriosis [fragments (b), (d)].

In the laser polarization-visualized microscopic images of blood plasma films of patients from group 1 [Figure 3, fragment (a)] and group 2 [fragment (b)], it is seen that, irrespective of the physiological state, the polycrystalline network of albumin proteins (cylindrical crystallites size) has a distinct optical anisotropy, detected in the intersection planes of the polarizer 4 and the analyzer 9 in a sufficiently similar set of coordinates from distributed areas of light and dark spots of varying intensity.

Analysis of a series of induced autofluorescence images of blood plasma samples from both groups [Figure 3, fragments (c), (d)] revealed different coordinate distributions of albumin and globulin autofluorescence proteins. Moreover, in the image of a plasma film taken from a patient in group 2, the laser-induced autofluorescence intensity is higher [fragment (d)] than the intensity propagation at the points of the corresponding image obtained from a sample in group 1 [fragment (c)].

It should be expected that the qualitatively analyzed autofluorescence manifestations of the tendency of change in the structure of polycrystalline protein networks of plasma films of patients from different groups would clearly show differences in statistical criteria characterizing the corresponding laser-induced Jones-matrix autofluorescence images.

### Experimental studies of the distributions of the Jones matrix elements of laser autofluorescence of protein networks of blood plasma films in the diagnosis of endometriosis

This section will analyze the possibilities of objective but indirect diagnosis of endometriosis by investigating the coordinate distributions of the right component of the Jones-matrix element of laser autofluorescence of protein networks of blood plasma film samples.

**Figure 4:**
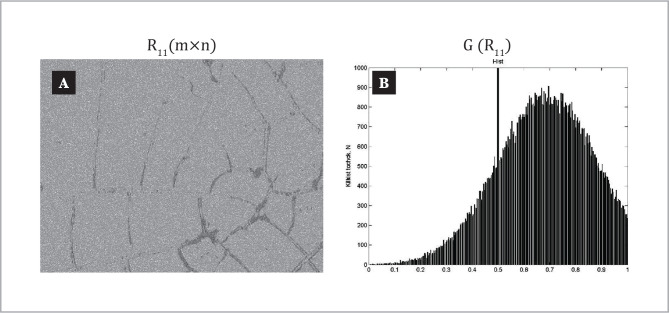
Coordinate () (a) and probabilistic (b) distributions of Jones matrix image of laser autofluorescence of blood plasma film in control group 1.

The coordinate distribution of the Jones matrix element and the histograms of its random values in the plane of the plasma film sample taken from patients in control group 1 are shown in the fragments of Figure 4.

Analysis of the coordinate distribution [Figure 4, fragment (a)] of the Jones-matrix image of laser autofluorescence of protein networks of the plasma film of a patient from control group 1 revealed a marked phenomenon of induced laser autofluorescence.

The structure of the histogram of the experimentally measured distribution of Jones-matrix element values in the plane of the plasma film consists of the main extremum and a sufficiently narrow range of change () of eigenvalues. [Figure 4, fragment (b)].

The dependence of the histogram is characterized by a clearly delineated sharp peak of the main extremum.

This feature can be attributed to the optically active polycrystalline network of albumin proteins and globulin of the plasma film taken from a healthy patient who sufficiently weakly absorbs laser radiation. As a result, there is a weak auto-fluorescence. This is indicated by the localization of values of random distribution in the region of transparency of a given fabric, manifested mainly by transformation mechanisms of polarization states of laser radiation by polycrystalline structures.

Quantitatively optical manifestations of laser-induced autofluorescence of protein molecules from the blood sample of a plasma film of a healthy patient from the control group illustrates a set of values of statistical moments of the 1^st^–4^th^ orders, which characterizes the coordinate distribution of an element of the Jones matrix (calculated by the relation 2.7). As it can be seen, the probability distribution of the random values of the element of the Jones matrix of laser autofluorescence of a sample of a plasma film taken from a patient in control group 1 is characterized by the advantage of the magnitude of the statistical moment of the 4^th^ order – M_i=4_^α^ ⪼ M_i=1;2,3_^α^, which determines the sharpness of the peak of the histogram G (R_11_).

In other words, for blood plasma taken from a patient with physiologically normal endometrial tissue, there is a slight level of intrinsic autofluorescence of albumin and globulin protein molecules, which is statistically manifested in the formation of an asymmetric (narrowly dissociated) 2, paragraph 2.5 of the distribution of the Jones element of the laser autofluorescence of the corresponding sample from control group 1.

Certain pathological changes in the human body manifest in changes in the biochemical composition of blood plasma proteins.

It is known that such processes are accompanied by an increase in the concentration of globulin and the level of absorption of laser radiation, which in turn induces the autofluorescence of such polycrystalline protein structures of the blood plasma film.

In our case, we should expect a certain increase in the laser autofluorescence intensity, which is quantitatively detected in the decrease in the relative values of the distribution of the element of the Jones plasma film matrix [section 3, paragraph 3.2, correlation (3.1)].

**Figure 5:**
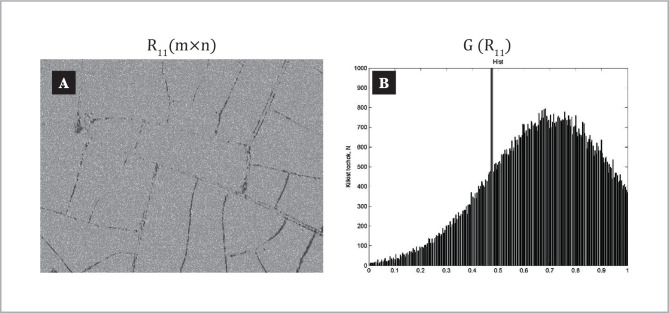
Coordinate () (a) and probabilistic (b) distributions of Jones matrix image of laser autofluorescence of polycrystalline protein network of blood plasma film taken from group 2.

Figure 5 shows the results of an experimental study of the coordinate distribution [fragment (a)] and the histogram of random values [fragment (b)] of the Jones matrix element of laser autofluorescence of polycrystalline proteins of plasma film proteins taken from a patient in group 2.

The experimental data on the structure of the coordinate [Figure 5, fragment (a)] and random (histogram) [Figure 5, fragment (b)] distributions of the magnitudes of the elements in the Jones matrix image revealed a certain increase in the intensity level of the induced laser autofluorescence of the protein molecules albumins and globulins forming a polycrystalline network in the sample of a patient in group 2.

The analysis of the histogram structure of the distribution of the Jones-matrix image of a plasma film from group 2 revealed a decrease in the level (peak acuity) of the main extremum and an increase in the dispersion of random values compared to the similar distribution of the random values of this statistical distribution determined for the plasma distribution taken from patients from control group 1 [Figure 4, fragment (b)].

This fact can be attributed to the processes of pathological changes in the endometrial layer, which indirectly manifest in the growth of laser radiation absorption by the globulin plasma of a diseased patient whose concentration has increased. Due to this biochemical phenomenon, the distribution of Jones-matrix image values with smaller relative values is formed, and the corresponding decrease in peak acuity and increase of dispersion of random values of the Jones-matrix element R_11_.

Quantitative changes in the biochemical structure of the molecules of protein crystalline networks in a sample of a plasma film taken from a patient in group 2 illustrates the increase (up to 20%) of the statistical moment of the 2^nd^ order and the decrease of the statistical moments of the 3^rd^ (up to 30%) and the 4^th^ orders (up to 35%).

These statistical parameters characterizing the coordinate structure of the Jones matrix image of laser autofluorescence of protein polycrystalline networks of albumin and globulin of the plasma film of a patient in group 2, acquire the following values – M_1_^α^ =0.9; M_2_^α^ =0.11; M_3_^α^ =0.31; M_4_^α^ =1.74.

Thus, it can be stated that the statistical analysis of the probabilistic distribution of random values of the true component of the Jones matrix element R_11_ m×n laser autofluorescence of plasma film protein molecules, as in the case of protein networks of histological sections of endometrial biopsy, showed sensitivity (albeit less) to changes in the physiological state of the person associated with endometriosis.

The following ranges of differences of average values are established M_i_^α^_=1;2;3;4_, that characterize the coordinate (m×n) and probabilistic G (R_11_) distributions of Jones-matrix images R_11_ m×n laser autofluorescence of polycritical networks of albumin and globulin of plasma films taken from patients in both groups (Table 1).

**Table 1:** Mean M_1_^α^ , dispercion M_2_^α^ , asymmetry M_3_^α^ and excess M_4_^α^ of distributions R_11_ (m×n) of blood plasma films taken from different groups.

M_k_^α^	Group 1 Norm (35 samples)	Group 2 Endometriosis (85 samples)
**M_1_^α^**	0.91±0.13	0.87±0.11
**M_2_^α^**	0.07±0.011	0.11±0.014
**M_3_^α^**	0.41±0.072	0.29±0.034
**M_4_^α^**	2.36±0.31	1.68±0.29

However, the statistical analysis of ranges of change in the magnitudes of statistical moments M_i_^α^_=1;2;3;4_ obtained from experimental studies of the statistical structure of the distributions of the elements of the Jones matrix laser autofluorescence in blood plasma films, as well as for samples of histological sections of the endometrium, within individual groups of healthy and sick patients, showed an increase in each group as false positive and negative.

Therefore, an intragroup analysis is relevant for determining the informativeness of the Jones-matrix mapping method for the autofluorescence of blood plasma films from patients in all groups.

## DISCUSSION

This section presents the results of determining a set of operating characteristics that determine the strength of the Jones-matrix method of laser autofluorescence of polycrystalline protein networks of blood plasma films in the diagnosis of endometriosis – sensitivity Se (relation; specificity Sp (relation; accuracy Ac (relation); predictive value of a positive result +VP (relation); the predictive value of a negative result -VP (ratio) of blood plasma film samples taken from patients with a reference diagnosis – control group 1 (35 samples) and group 2 (85 samples).

The results objectivity of the physical method of Jones-matrix mapping was achieved by a complex computer analysis of the statistical structure of autofluorescence intensity maps of digital microscopic images of blood plasma films based on the calculation of blood plasma 48,000 pixels of set from 1^st^ to 4^th^ order stats M_k_^α^_=1;2;3;4_.

Each of these parameters U^α^ ⇔ M_k_^α^_=1;2;3;4_ is an object for evaluating the strength of a method U^α^ ⇔ {Se Sp Ac+VP-VP} based on the formation of a matrix of decisions according to the application of the Jones-matrix method of autofluorescence mapping of polycrystalline networks of plasma proteins taken from patients in all groups with a pre-established verified (reference) diagnosis.

The analysis of the obtained data revealed the following: the strength of the Jones-matrix method of autofluorescence mapping of plasma protein proteins taken from patients in group 1 and group 2 is maximal for the decisions determined based on the calculation of the statistical moment of the 4^th^ order. For statistical moment Z_4_^α^ , characterizing the sharpness of the peak distribution of the polycrystalline component of the plasma film, the number of positive and false negative solutions was 69 and 26 (sensitivity Se=87%), and the number of negative and false positive decisions was 25 and 10 (specificity Sp=74%). For the statistical moment Z_3_^α^ , which characterizes the asymmetry of the distribution of the polycrystalline component of blood plasma films, the number of positive and false negative solutions was 77 and 8 (sensitivity Se=81%), and the number of negative and false positive decisions was 27 and 8 (specificity Sp=71%); the accuracy of the method Ac was 76%.

Thus, it is possible to ascertain the proper level of reliability or validity of the method, which demonstrated the correspondence of the obtained set of statistical parameters characterizing the Jones-matrix distributions of the laser autofluorescence intensity of polycrystalline networks of plasma films to the specific condition of the patient.

## Conclusion

Comparison with similar informative data of the Jones-matrix laser autofluorescence method of histological sections of the endometrial biopsy under conditions of blind endometriosis diagnosis rendered this method relatively high (although 15%–25% lower) informative. Subsequently, this technique should be used in screening studies to form a risk group for appearing endometriosis.

## Data Availability

All raw data of the study are available by request.
